# Mechanism of axillary nerve palsy in the terrible triad of the shoulder: the role of excessive dislocation

**DOI:** 10.1016/j.jseint.2026.101669

**Published:** 2026-02-11

**Authors:** Yoshihiro Nakamura, Toshiyuki Fukushima, Takahiko Hamasaki, Eisaku Fujimoto, Yohei Harada, Nobuo Adachi

**Affiliations:** a Department of Orthopaedic Surgery, Chugoku Rosai Hospital, Hiroshima, Japan; b Central Department of Rehabilitation, Chugoku Rosai Hospital, Hiroshima, Japan; c Department of Orthopaedic Surgery, Hiroshima University, Hiroshima, Japan

**Keywords:** Anterior shoulder dislocation, Closed reduction, Hill–Sachs lesion, Nerve palsy, Rotator cuff tear, Shoulder, Terrible triad of the shoulder

## Abstract

**Background:**

The “terrible triad of the shoulder” (TTS) is a rare condition involving the simultaneous occurrence of shoulder dislocation, rotator cuff tear, and nerve palsy. The mechanism underlying its development remains unclear. This study aimed to examine the dislocation characteristics in patients with TTS.

**Methods:**

Eight patients with primary anterior shoulder dislocation, electromyography-confirmed axillary nerve palsy, and magnetic resonance imaging–verified rotator cuff tears were classified as the TTS group. Another 8 patients with primary anterior shoulder dislocation but without clinical evidence of brachial plexus palsy served as the control group. On anteroposterior radiographs taken during dislocation, the distance between the inferior glenoid margin and the medial anatomical neck of the humerus was measured as the dislocation distance. The superolateral tip of the greater tuberosity was assessed as being medial or inferior to the glenoid. The presence of Hill–Sachs lesions was evaluated on postreduction computed tomography scans.

**Results:**

The dislocation distance was significantly greater in the TTS group than in controls (49.3 ± 4.4 mm vs. 33.3 ± 3.6 mm, *P* = .0009). The greater tuberosity was positioned medial or inferior to the glenoid during dislocation in 75% of TTS cases and 0% of controls (*P* = .007). Hill–Sachs lesions were absent in all TTS cases, whereas they were observed in 62.5% of the control group (*P* = .026).

**Conclusion:**

Excessive displacement in anterior shoulder dislocation with rotator cuff tear may be associated with axillary nerve traction. Imaging performed at the time of dislocation may help predict the occurrence of TTS.

Shoulder dislocation can occur with brachial plexus palsy, often involving the axillary nerve.^1^^,^^10^^,^^17^^,^^20^^,^^23^ Previous reports have shown that dislocations accompanied by rotator cuff tears are more likely to result in nerve palsy.^1^^,^^20^ The simultaneous occurrence of shoulder dislocation, nerve palsy, and rotator cuff tear is referred to as the terrible triad of the shoulder (TTS).^4^^,^^7^ However, the mechanism behind the higher likelihood of nerve palsy in shoulder dislocations with rotator cuff tears has not been clearly investigated. In anterior shoulder dislocations, the posterior humeral head typically impacts the anterior glenoid rim, producing a Hill–Sachs lesion medial to the infraspinatus tendon insertion. When a rotator cuff tear is present, however, the humeral head may dislocate beyond the typical site of a Hill–Sachs lesion, causing greater displacement and excessive traction on the axillary nerve, which may result in palsy. Therefore, this study aimed to clarify the dislocation characteristics associated with TTS. We hypothesized that TTS cases are characterized by excessive displacement at the time of dislocation.

## Patients and methods

This study was approved by the institutional ethics committee (2025-10). We retrospectively reviewed 11 patients diagnosed with the TTS between 2019 and 2024. All cases involved a first-time anterior shoulder dislocation; 1 case was spontaneously reduced, and the remaining was treated with closed reduction. All patients were immobilized in adduction and internal rotation using a sling and body band for three weeks. After immobilization removal, all patients continued to experience difficulty with active shoulder elevation. Magnetic resonance imaging (MRI) confirmed rotator cuff tears in each case. Needle electromyography revealed positive sharp waves and fibrillation potentials in the deltoid muscle, consistent with axillary nerve palsy, confirming the diagnosis of TTS. Two patients without radiographs at the time of dislocation and 1 whose dislocation was spontaneously reduced before radiographic examination were excluded. Consequently, 8 patients with TTS and available anteroposterior radiographs taken during dislocation were included (mean age, 76.0 ± 10.0 years; 6 men and 2 women). The control group included 8 patients (mean age, 59.4 ± 29.0 years; 3 men and 5 women) who underwent closed reduction for first-time anterior shoulder dislocation in 2024, had radiographs obtained at the time of dislocation, and showed no clinical signs of brachial plexus palsy. In the control group, MRI, ultrasonography, and electrophysiological examinations were not routinely performed.

**Figure 1:**
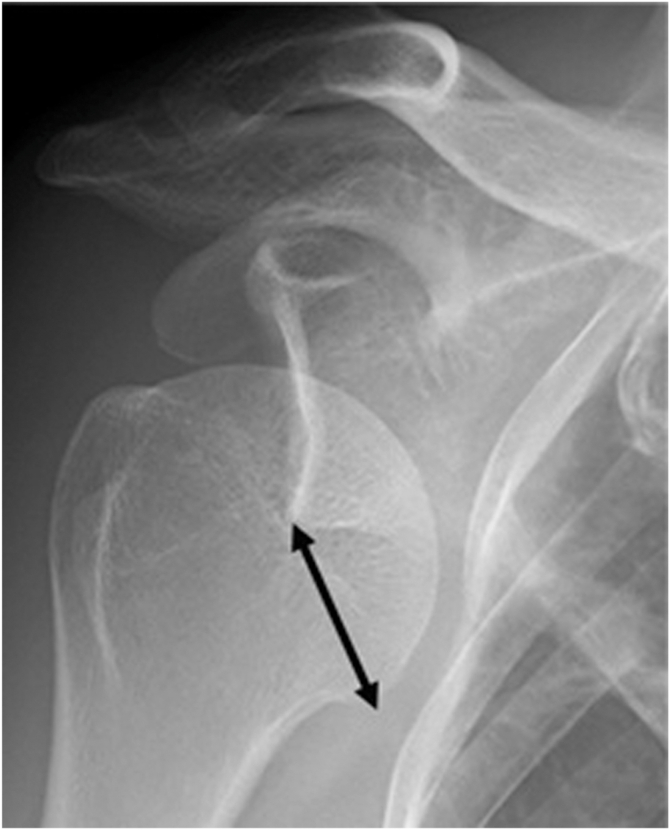
Measurement of dislocation distance on anteroposterior radiographs taken at the time of dislocation. The bidirectional arrow represents the distance between the inferior glenoid margin and the medial aspect of the humeral anatomical neck, defined as the dislocation distance.

**Figure 2:**
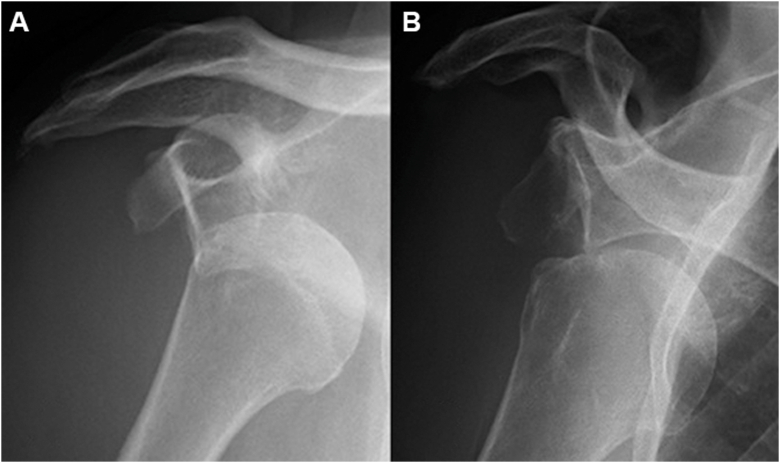
Representative anteroposterior radiographs taken at the time of dislocation showing the position of the greater tuberosity relative to the glenoid. (**A**) The greater tuberosity is positioned medial to the glenoid. (**B**) The greater tuberosity is positioned inferior to the glenoid.

The following variables were evaluated: time from dislocation to reduction, use of brachial plexus block or sedation during reduction, and presence of Hill–Sachs lesions on postreduction computed tomography. On anteroposterior radiographs taken during anterior shoulder dislocation, we measured the distance between the inferior glenoid margin and the medial aspect of the humeral anatomical neck (Fig. 1) and assessed whether the superolateral tip of the greater tuberosity was positioned medial to or inferior to the glenoid (Fig. 2). Cases showing either medial or inferior positioning were classified as having excessive displacement.

These parameters were compared between the TTS and control groups. The Mann–Whitney *U* test was used to assess differences in age, time to reduction, and dislocation distance between groups, given the small sample size and the inability to assume a normal distribution. Fisher exact test was applied to categorical variables, including sex, use of brachial plexus block or sedation, presence of Hill–Sachs lesions, and medial or inferior positioning at dislocation. A *P* value <.05 was considered statistically significant. All statistical analyses were performed using EZR (Saitama Medical Center, Jichi Medical University, Saitama, Japan), a graphical user interface for R (The R Foundation for Statistical Computing, Vienna, Austria).

## Results

**Figure 3:**
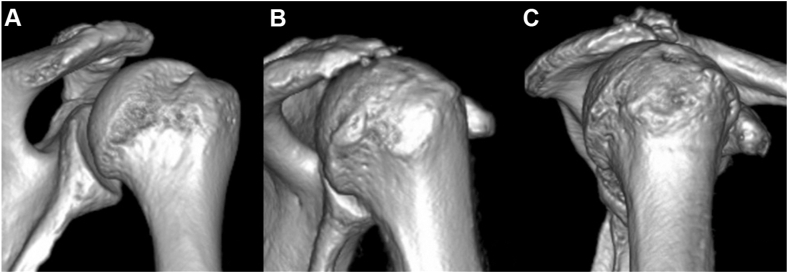
Evaluation of Hill–Sachs lesions on CT scans obtained after reduction. (**A**) Typical Hill–Sachs lesion in the control group. (**B**) Absence of a Hill–Sachs lesion in the TTS group. (**C**) Impaction at the middle facet of the greater tuberosity in the TTS group. *CT*, computed tomography; *TTS*, terrible triad of the shoulder.

All 8 patients with the TTS sustained injuries from low-energy trauma and underwent closed reduction within 24 hours. All cases involved supraspinatus and infraspinatus tendon tears, and 4 also had subscapularis tendon tears. Fatty infiltration of the supraspinatus was advanced, with 7 patients graded ≥ 3 according to the modified Goutallier classification.^3^^,^^6^ Five patients showed a modified Goutallier grade ≥ 3 in the infraspinatus. In addition to axillary nerve palsy, 3 patients had suprascapular nerve palsy, and one of these also exhibited musculocutaneous, median and ulnar nerve palsies. No Hill–Sachs lesions were identified in any TTS cases; instead, 3 patients demonstrated impaction at the middle facet of the greater tuberosity (Fig. 3 and Table I). At the time of diagnosis of TTS, no patient showed clinical signs or reported subjective symptoms of shoulder instability. One patient experienced severe shoulder pain, whereas all presented with difficulty in active shoulder elevation as the chief complaint. Nerve palsy was managed conservatively with observation, anticipating spontaneous recovery. No rotator cuff repairs were performed. All patients underwent rehabilitation, and analgesics were prescribed as needed. Signs of neurological recovery appeared at an average of 3 months after dislocation (range, 1-5 months). Three patients underwent reverse total shoulder arthroplasty (rTSA) for irreparable rotator cuff tears that caused persistent difficulty with active shoulder elevation despite showing signs of neurological recovery. rTSA was performed an average of 4.7 months after dislocation (range, 3-6 months), and these patients achieved active shoulder flexion greater than 90° in the sitting position at an average of 2.3 months post-operatively (range, 1-4 months). The remaining 5 patients continued conservative management; 4 achieved active flexion >90° in the sitting position at an average of 7.5 months after dislocation (range, 4-14 months). One patient remained unable to actively elevate the arm after one year but declined surgery. Ultimately, none of the 8 patients had residual pain requiring further treatment (Table II).

**Table I:** Clinical characteristics of 8 cases of the terrible triad of the shoulder.

Case	Age/sex	Injury mechanism	Time to reduction (h)	RCT	FI			Nerve palsy	HSL
					SSC	SSP	ISP		
1	67/M	Fall from bed	20	SSP, ISP	0	1	1	AxN, SSN, MCN, MN, UN	-
2	59/M	Fall from a height of 1 meter	6	SSC, SSP, ISP	1	3	2	AxN	-
3	74/M	Standing fall	12	SSP, ISP	0	4	4	AxN	-∗
4	75/F	Standing fall	3	SSP, ISP	1	4	4	AxN, SSN	-
5	78/M	Standing fall	1	SSC, SSP, ISP	4	4	4	AxN, SSN	-∗
6	83/M	Standing fall	3	SSC, SSP, ISP	2	4	3	AxN	-
7	92/M	Fall from a height of 1 meter	3	SSP, ISP	1	3	2	AxN	-∗
8	80/F	Standing fall	3	SSC, SSP, ISP	4	4	4	AxN	-

*M*, male; *F*, female; *RCT*, rotator cuff tear; *SSP*, supraspinatus; *ISP*, infraspinatus; *SSC*, subscapularis; *FI*, fatty infiltration; *AxN*, axillary nerve; *SSN*, suprascapular nerve; *MCN*, musculocutaneous nerve; *MN*, median nerve; UN, ulnar nerve; *HSL*, Hill–Sachs lesion.

∗ Impaction at the middle facet was observed.

**Table II:** Clinical course after diagnosis in patients with the terrible triad of the shoulder.

Case	Age/sex	Time of diagnosis(mo)	Pain at diagnosis	Treatment	Time to sign of nerve recovery (mo)	Time to achieve active flexion >90° (mo)	Follow-up period (mo)	Final follow-up				
								VAS (points)	Flex	Abd	ER	IR
1	67/M	1	None	Conservative	1	5	6	0	110	100	50	L1
2	59/M	2	None	Conservative	2	14	39	1	100	80	70	L2
3	74/M	1	None	rTSA (3 mo)	2	7 (4 after rTSA)	12	0	95	100	15	L4
4	75/F	2	Mild	rTSA (6 mo)	4	8 (2 after rTSA)	67	0	115	105	30	S
5	78/M	2	None	rTSA (5 mo)	4	6 (1 after rTSA)	55	0	155	135	40	L2
6	83/M	1	Severe	Conservative	5	-	12	2	30	30	10	S
7	92/M	2	None	Conservative	3	4	12	0	110	105	0	L1
8	80/F	1	None	Conservative	3	7	24	0	135	85	0	L1

*rTSA*, reverse total shoulder arthroplasty; *VAS*, visual analog scale; *Flex*, flexion; *Abd*, abduction; *ER*, external rotation; *IR*, internal rotation, expressed as the spinal level reached by the thumb behind the back (L = lumbar spine, S = sacral spine).

When comparing the TTS group with the control group, there were no significant differences in age, sex, time to reduction, or use of brachial plexus block or sedation during reduction. Hill–Sachs lesions were present in 5 patients (62.5%) in the control group but in none of the TTS cases (*P* = .026). In the TTS group, 75% of cases showed the superolateral tip of the greater tuberosity positioned medial or inferior to the glenoid at dislocation (excessive displacement), compared with 0% in the control group (*P* = .007). Furthermore, the mean dislocation distance was significantly greater in the TTS group (49.3 ± 4.4 mm) than in the control group (33.3 ± 3.6 mm, *P* = .0009) (Table III).

**Table III:** Demographic and radiographic comparison between the terrible triad of the shoulder and control groups.

Variable	Terrible triad of the shoulder group (n = 8)	Control group (n = 8)	*P* value
Age (yr)	76.0 ± 10.0	59.4 ± 29.0	.38
Sex (male, female)	75.0%, 25.0%	37.5%, 62.5%	.32
Time to successful reduction (h)	6.4 ± 6.5	7.2 ± 11.7	.96
Brachial plexus block or sedation	37.5%	50.0%	1.00
Hill–Sachs lesion	0%	62.5%	**.026**
Medial position	37.5%	0%	.20
Inferior position	37.5%	0%	.20
Medial position or inferior position	75.0%	0%	**.007**
Dislocation distance (mm)	49.3 ± 4.4	33.3 ± 3.6	**.0009**

Bold values indicate statistical significance (*P* < .05).

## Discussion

Based on the findings of the present study, anterior dislocation in the TTS was associated with excessive displacement and absence of Hill–Sachs lesions. When a rotator cuff tear is present, excessive displacement may be more likely, thereby potentially increasing the risk of nerve palsy.

**Figure 4:**
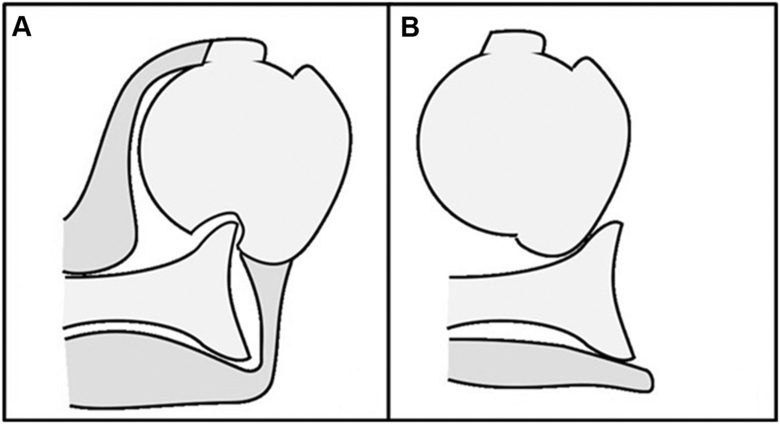
Schematic illustration of anterior shoulder dislocation patterns. (**A**) Typical anterior shoulder dislocation showing formation of a Hill–Sachs lesion on the posterolateral aspect of the humeral head. (**B**) Anterior shoulder dislocation with a concomitant rotator cuff tear, demonstrating excessive displacement without formation of a Hill–Sachs lesion.

Nerve palsy has been reported as a complication after anterior shoulder dislocation.^1^^,^^10^^,^^17^^,^^20^^,^^23^ Robinson et al^20^ reported nerve palsy rates of 8.7% in dislocations without rotator cuff tears or greater tuberosity fractures, 23.3% in cases with either rotator cuff tears or greater tuberosity fractures, 17.2% in those with rotator cuff tears alone, and 26.8% in those with greater tuberosity fractures, indicating a higher incidence when such injuries are present. These variations may reflect different dislocation mechanisms. The primary mechanism of nerve palsy after shoulder dislocation is believed to be traction injury, as motor deficits typically exceed sensory deficits.^9^ The axillary nerve, which runs close to the shoulder joint, is especially vulnerable to traction; consequently, axillary nerve palsy is the most common nerve injury associated with anterior shoulder dislocation.^1^^,^^17^^,^^20^^,^^23^ Hill–Sachs lesions have been observed in 58%-93% of first-time anterior shoulder dislocations.^12^^,^^16^^,^^19^ In this study, Hill–Sachs lesions were found in 62.5% of the control group, aligning with prior reports, but were absent in all TTS cases. Instead, several patients showed impaction at the middle facet. This finding suggests a dislocation mechanism distinct from that of typical anterior shoulder dislocations, in which the posterior humeral head impacts the anterior glenoid rim. Moreover, in the TTS group, radiographs taken during dislocation often showed the greater tuberosity positioned medial or inferior to the glenoid, indicating greater displacement. These observations support the hypothesis that TTS cases involve excessive displacement at the time of dislocation. Collectively, these findings suggest that in anterior shoulder dislocations with rotator cuff tears, the humeral head may dislocate beyond the typical site of a Hill–Sachs lesion medial to the infraspinatus tendon insertion, which may be associated with excessive displacement and subsequent nerve traction (Fig. 4).

Another key consideration is whether the rotator cuff tears observed in TTS were acute or preexisting. In this study, many patients showed advanced fatty infiltration of the supraspinatus and infraspinatus, suggesting that most tears were chronic.^14^^,^^15^ Therefore, pre-existing rotator cuff pathology may predispose patients to excessive displacement and contribute to nerve palsy development. Regarding the relationship between time to reduction and nerve palsy, some studies have reported that longer delays increase risk, whereas others found no correlation. In this study, comparing TTS cases with dislocations without nerve palsy revealed no significant difference in time to reduction.

Gumina et al^8^ reported axillary nerve palsy in 9.3% of anterior shoulder dislocations, with full recovery within 3-12 months. Likewise, previous research has shown spontaneous recovery of nerve palsy even in TTS cases, supporting conservative observation as the initial management approach.^2^^,^^5^^,^^7^^,^^13^^,^^21^^,^^22^ However, several authors have cautioned that during the recovery period, muscle atrophy and fatty degeneration may progress, complicating rotator cuff repair. Thus, early rotator cuff repair has been advocated.^2^^,^^5^^,^^7^^,^^13^^,^^21^^,^^22^ Nonetheless, outcomes for massive rotator cuff tear repairs are often poor.^13^^,^^21^ For irreparable tears, staged approaches such as arthroscopic superior capsule reconstruction after nerve recovery^11^ or staged reverse end-to-side radial-to-axillary nerve transfer followed by rTSA after recovery of deltoid function^18^ have been described. In this study, 3 patients underwent rTSA for irreparable rotator cuff tears after showing signs of neurological recovery, achieving satisfactory range of motion. In contrast, there has also been a report of a favorable outcome with conservative treatment for both nerve palsy and rotator cuff tears.^7^ In this study, among the 5 patients managed conservatively, 4 achieved active flexion >90° within 4-14 months, whereas 1 failed to elevate the arm after 1 year. Taken together, these findings suggest that conservative observation remains the mainstay for nerve palsy in TTS. Early surgical repair is advised for reparable rotator cuff tears, whereas for irreparable tears, initial conservative management with close monitoring of nerve recovery is recommended. If persistent elevation disability remains after nerve recovery, staged rTSA or, for younger patients, arthroscopic superior capsule reconstruction may be considered effective. Establishing this treatment strategy requires early diagnosis of TTS using MRI and electrophysiological examinations after dislocation. Careful evaluation at the time of dislocation is therefore crucial to avoid missed diagnoses. Based on the present findings, when radiographs show marked humeral head displacement, clinicians should suspect a concomitant rotator cuff tear and consider the possibility of TTS with associated nerve palsy.

This study has several limitations. First, it was retrospective in nature and included a small number of patients. Because of the limited sample size, multivariable regression analysis could not be performed to adjust for potential confounding factors. As a result, differences in baseline characteristics between groups, including age, sex distribution, and the use of brachial plexus block or sedation during reduction, may have influenced the results. Second, the dislocation distance was measured on anteroposterior radiographs. This method has inherent limitations and does not allow accurate assessment of three-dimensional displacement or axillary nerve traction. In addition, measurements were performed by a single observer, and interobserver or intraobserver reliability was not evaluated. Third, because TTS is rare and most cases were referred from other facilities, the proportion of TTS among all anterior shoulder dislocation cases in the cohort was unknown, and direct comparison with all dislocation cases was not possible. Finally, MRI, ultrasonography, and electrophysiological examinations were not performed in the control group, which may have led to underdiagnosis of mild nerve injuries or rotator cuff pathology and may have affected the validity of comparisons between groups.

## Conclusion

In TTS, anterior dislocation was associated with excessive humeral head displacement and absence of a Hill–Sachs lesion. Such displacement may contribute to traction injury of the axillary nerve. Recognizing marked displacement on radiographs at the time of dislocation may be helpful for early suspicion of TTS and for informing subsequent treatment planning.

## Disclaimers:

Funding: No external funding was received for this study.

Conflicts of interest: The authors declare that they have no conflicts of interest relevant to this study.
